# Preparation of poly glycidyl methacrylate (PGMA) chain-grafted boron nitride/epoxy composites and their thermal conductivity properties†

**DOI:** 10.1039/d1ra00976a

**Published:** 2021-06-24

**Authors:** Haibao Zhang, Xian Zhang, Kang Zheng, Xingyou Tian

**Affiliations:** Institute of Solid Physics, Hefei Institutes of Physical Science, Chinese Academy of Sciences Hefei People's Republic of China hbzhang@rntek.cas.cn; Key Lab of Photovoltaic and Energy Conservation Materials, Institute of Solid State Physics, HFIPS, Chinese Academy of Sciences Hefei 230031 China xzhang@issp.ac.cn

## Abstract

Surface modification of hexagonal boron nitride (h-BN) has the problem of reducing the interfacial thermal resistance, which has hindered its application in thermal conductive composites. Herein, poly glycidyl methacrylate (PGMA) chains were grafted onto the h-BN surface by simple radical polymerization; the thermal conductivity of epoxy (EP) composites was improved by adding the as-grafted h-BN–PGMA to EP resin. When the filling volume of h-BN–PGMA was 4, 10 or 16 vol%, the thermal conductivity of EP composite increased by 160%, 298% or 599%, respectively. Moreover, the h-BN surface modification was beneficial to enhance the compatibility between the filler and the EP matrix. Compared to EP/h-BN, the EP/h-BN–PGMA had higher thermal conductivity (1.197 W m^−1^ K^−1^) under the same filling amount (16 vol%). Moreover, excellent dielectric properties and thermal stability indicated that EP/h-BN–PGMA composites were excellent thermal interface materials (TIMs) and could be applied in the field of thermal management. The preparation process is environmentally friendly, easy to operate, and suitable for large-scale practical applications.

## Abstract 2

A schematic illustration of the preparation process of PGMA chain grafted h-BN.

## Graphical abstract

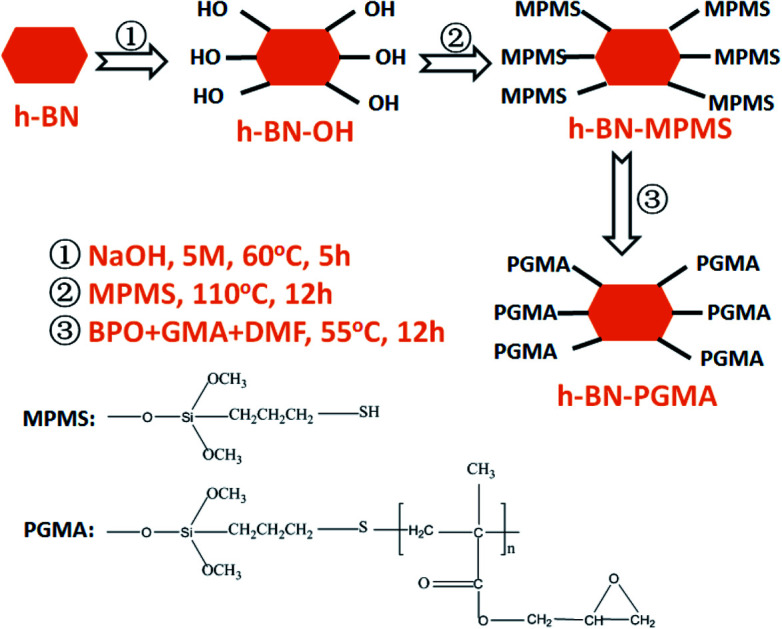

## 1. Introduction

Epoxy resin (EP) is widely used in the process of electronic packaging substrates, which makes the electronic devices exhibit long service life and good performance.^1^ However, its thermal conductivity is low (∼0.2 W m^−1^ K^−1^), and the packaging process is complex, which is not beneficial to its further development. With the development of the next generation microelectronic technology, thermal management has become a key link in the production of high thermal conductivity insulating electronic packaging materials.^2^ The high-efficiency heat transfer of polymer composites with high thermal conductivity and low conductivity fillers has been paid more and more attention in the thermal management materials field.^3^ A thermal interface material (TIMs) is a polymer-based material that is commonly used in electronic devices to reduce operating temperature and support optimal performance and service life.^4^ The design and fabrication of epoxy-based electronic packaging materials with excellent thermal conductivity (greater than 1 W m^−1^ K^−1^) remain challenging.^5^ Therefore, how to effectively improve the thermal conductivity of epoxy resin is particularly important in electronic packaging technology.

It is well known that the incorporation of highly thermally conductive ceramic particles such as aluminum nitride (AlN),^6^ alumina (Al_2_O_3_),^7^ silicon carbide (SiC),^8^ silicon nitride (Si_3_N_4_)^9^ into the polymer matrix can greatly improve the thermal conductivity of composites. However, high thermal conductivity often requires high content fillers to establish a heat conduction network. However, the high filling amount will decrease the mechanical properties and dielectric properties of the composites. Hexagonal boron nitride (h-BN) is the most commonly used heat conduction filler, because h-BN has very high thermal conductivity (250–300 W m^−1^ K^−1^), very low dielectric constant, while the thermal conductivity of traditional ceramic fillers (such as Al_2_O_3_, AlN) is far less than that of h-BN. Although the thermal conductivity of h-BN is much lower than that of graphene, its high resistance and ideal dielectric properties make it a feasible candidate in the electronic packaging technology field.^10^ We often use h-BN filler to prepare thermal conductivity and electrical insulation composite materials. Because it has good thermal conductivity with in-plane thermal conductivity ranging from 300 W m^−1^ K^−1^ to 600 W m^−1^ K^−1^, and it has also good electrical insulation.^11^ Furthermore, the two-dimensional h-BN filler has anisotropy, and when it is added to the polymer matrix, the thermal conductivity of this composite is also anisotropic.^12^ The orientation of BNNS is horizontal in-plane direction,^13^ and the in-plane thermal conductivity of the composite is obviously higher than that of the through-plane surface. The modified boron nitride is more widely used and has an excellent performance in many aspects, it is usually modified by acid–base composite materials. Lin *et al.*^14^ functionalized h-BN fillers with octadecyl amine (ODA) or oligomeric terminal amino polyethylene glycol (PEG) to obtain ODA-BN or PEG-BN functionalized fillers. *Via* the interaction of amino groups with boron atoms, when the mass fraction of the as-functionalized h-BN filling amount was 75 wt%, the h-BN-based polyimide (PI) composites were prepared by Sato *et al.*,^15^ while the thermal conductivity of PI composites was as high as 7 W m^−1^ K^−1^. Chen *et al.*^16^ constructed a three-dimensional aerogel (3D-BNNS) thermal network in the epoxy resin (EP) matrix and filled the EP matrix. The study found that the thermal conductivity of EP/3D-C-BNNS composites could be increased by about 14 times when the 3D-BNNS filling amount was 9.6 vol%. The increase in thermal conductivity was due to the formation of a thermal network. Nano-fibrous inorganic fillers were easy to disperse in the polymer matrix due to their large aspect ratio, and could also establish an effective thermal conductivity network at low filling capacity. At the same time, grafting polymers on the surface of thermal conductive particles have been shown to be an effective method to improve the dispersion of fillers and enhance the compatibility between the fillers and the polymer matrix, which would lead to an increase in thermal conductivity of composites.^17^

The data in Table 1 could be seen that the different filling amounts of fillers and the different methods of testing thermal conductivity would affect the thermal conductivity and dielectric properties of composites. The filling amount of functionalized BN filler in the table is the optimal content. The results showed that the AgNPs were used as an adhesive to bridge two adjacent BNNS to form a continuous thermal conductivity pathways. At this time, the thermal conductivity of the composite was 1330% times that of pure epoxy resin (EP = 0.23 W m^−1^ K^−1^). Nevertheless, AgNPS particles were easy to agglomerate, oxidize easily, and had instability. All the above functionalized BN fillers had high filling capacity, and the thermal conductivity of the composites was also improved, but the mechanical properties of the composites were reduced. The morphology of fillers had special requirements. The base-EP composites prepared by blending epoxy resin and BN filler or modified BN filler usually showed lower thermal conductivity than EP/h-BN–PGMA composites in this work, except for EP/BN/AgNPs composite.^18^ Therefore, through the various studies shown in Table 1, the h-BN–PGMA filler with a filling amount of less than 18 vol% was finally selected for ideal fillers. In this work, we selected a large aspect ratio h-BN to construct a thermal network with a low percolation threshold and established multiple PGMA macromolecular chain growth active points to achieve an enhancement of the interfacial interaction between h-BN and EP resin, and to improve the thermal conductivity of the EP matrix composites.

**Table 1:** Main parameters of various epoxy composites

Materials	Filler loading (vol%)	TC^b^ (W m^−1^ K^−1^)	Dielectric constants	Test method of TC^b^	Ref.
EP/BNNS	12^a^	0.387	—	Laser flash	4
EP/3D-C-BNNS	9.6	3.13	—	Laser flash	16
EP/BN-ODA	3^a^	0.329	—	Laser flash	14
EP/BN-HBP	6	0.246	4.45	Laser flash	28
EP/BN	18^a^	1.037	—	Hot wire	26
EP/BN	36^a^	1.05	6.55	TPS	27
EP/3D-BNNS	9.3	2.85	—	Laser flash	29
EP/aligned BN	44	9		Laser flash	32
EP/aligned BN	40	5.2		Laser flash	35
EP/BN/AgNPs	25.1	3.06		Laser flash	33
EP/BN	17.5	0.91	—	Laser flash	31
EP/h-BN–PGMA	16	1.198	3.42	Hot disk	This work

a Converting for mass fraction to volume fraction.

b TC is abbreviation of thermal conductivity.

## 2. Experiment

### 2.1 Material

h-BN powder (size is ∼15 μm, thickness is 200 nm, the aspect ratio is 150) was purchased from Dandong Rijin co., ltd (china), solvent: toluene (analytical pure AR) was purchased from Wuxi Yasheng chemical co., ltd, silane coupling agent, DMF, BPO and GMA were purchased from Shanghai Aladdin reagent co., ltd, deionized water was made in the laboratory.

### 2.2 Functionalization of h-BN surfaces

**Fig. 1:**
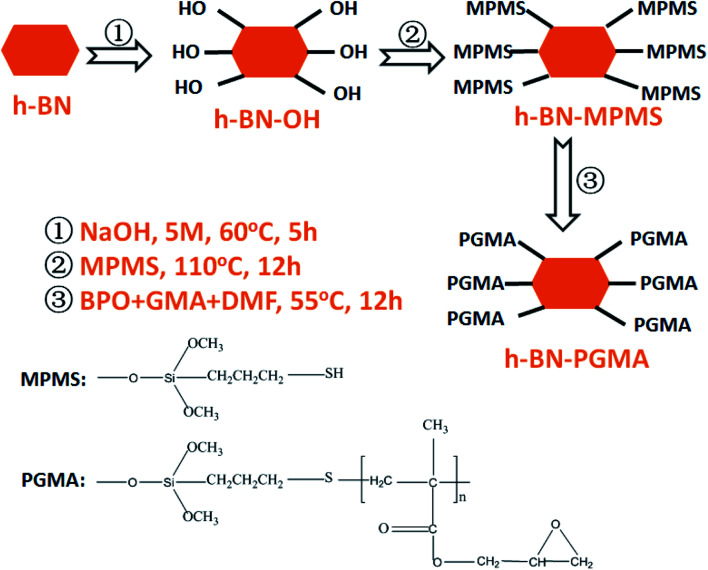
Preparation process of PGMA chain grafted h-BN.

According to previous reports, the surface hydroxylation of h-BN was achieved by interacting with a strong base.^19^ The edge surfaces of the h-BN successfully generated hydroxyl groups bound to boron atoms. According to Fig. 1, the synthesized hydroxylated h-BN (h-BN–OH) was grafted onto the coupling agent. The specific processes were as follows: h-BN–OH (2 g) and the appropriate amount of MPMS (2 wt% relative to the h-BN–OH) ultrasonically dispersed for 20 min in toluene solution (250 ml).^20^ The resulting suspension was refluxed and stirred for 10 h under nitrogen atmosphere conditions, and washed with multiple filtration to obtain grafted h-BN (h-BN–MPMS). Finally, the grafted h-BN (h-BN–PGMA) was prepared by simple radical polymerization through polymer-grafting modification. Construction of the experimental device: a 500 ml three-necked flask was fixed in a device equipped with a mechanical stirrer and a condensation reflux tube, 1 g of h-BN–MPMS and 10 ml of GMA were placed in 200 ml of DMF solvent, the obtained dispersion liquid was heated to 55 °C, and the solution was stirred under N_2_ atmosphere, then 0.083 g of weighed BPO was slowly added to the above reaction device, the reaction was continued for 12 h, and finally the as-prepared h-BN–PGMA was obtained (as shown in Fig. S1 and S3†), and subsequently dispersed in ethanol for later use.

### 2.3 Formulation design and preparation of epoxy composites

According to the above preparation process, h-BN or h-BN–PGMA suspensions (dispersed in ethanol) were mixed with an appropriate amount of epoxy resin. The excess ethanol after mixing could be removed by oven curing. The curing agent of 8 wt% quality (compared to epoxy resin) was added to the resulting blend and stirred for 1 h, and poured into the silica gel mold to cure by three gradient temperatures. The disc specimen with a diameter of 20 mm, a thickness of 2 mm was finally fabricated. h-BN–PGMA volume fraction contents in the composites were 4, 10 and 16 vol%, respectively, and the final epoxy composite block material could be directly used for the thermal conductivity test. EP/h-BN–PGMA composites were prepared by the same process as that for EP/h-BN.

### 2.4 Characterization instruments

X-ray film and powder diffraction (XRD) analysis were carried on a Philips X'Pert Pro MPD X-ray diffractometer (40 kV, 40 mA) with Cu-Kα radiation (*λ* = 0.1549 nm). The surface and cross-sectional morphology of the composite sample was observed using scanning electron microscope (Sirion 200 FESEM, FEI, USA), the acceleration voltage of the electron microscope was 10 kV at this time, all samples were coated with an Au-seed layer with a certain thickness in the ion-sputtering coater before observation. The dispersion of fillers in the composite was characterized using a transmission electron microscope (TEM) (JEM-2100, JEOL Electronics, Japan) at an acceleration voltage of 100 kV. The prepared powder was placed into ethanol for ultrasonication, then it was dropped on the copper mesh and dried for the next detection. Infrared spectrum analysis (NEXUS type) was conducted by mixing the sample and KBr, followed by grinding in a ratio of 1 : 120, the mixed powder was compressed and then taken out, and the sample was placed in an infrared instrument for testing. Thermogravimetric analysis (TGA) test: a thermogravimetric analyzer (TGA; Pyris 1, Perkin-Elmer, USA) was used for studying the thermal properties of the material under the nitrogen environment, the temperature was between 50 °C and 700 °C, and the heating rate was 10 °C min^−1^. A dynamic thermo-mechanical analyzer (Pyris Diamond DMA, Perkin Elmer Instruments, USA) was used for investigating the storage modulus (*G*′), loss modulus (tan *δ*) and glass transition temperature of the composites. Small strips sample with a size of 3 cm × 0.5 cm × 0.2 cm was detected at four different frequencies (0.1, 0.5, 1, 5 Hz). Differential thermal analyses were performed on a differential scanner (DSC Q2000 V24.10 Build 122, Shanghai Yukon Industrial co., ltd), under N_2_ atmosphere, and the temperature range was 50–250 °C, and the heating rate was 20 °C min^−1^. Waterproof detection was performed using a contact angle analyzer analysis (CAD-100, Shanghai Yingnuo Instrument co., ltd); the test was carried out with a 5 μl water drop sample. The chemical structures of the raw materials h-BN and h-BN–PGMA were determined using a FTIR spectrometer. The morphologies of h-BN and h-BN–PGMA were observed and compared using SEM and TEM. Their composition information was obtained by energy-dispersive X-ray spectroscopy (EDS, JEM-2010) (the performance of composite materials was characterized in the International Unit System).

## 3. Results and discussion

### 3.1 Surface treatment of thermally conductive fillers

**Fig. 2:**
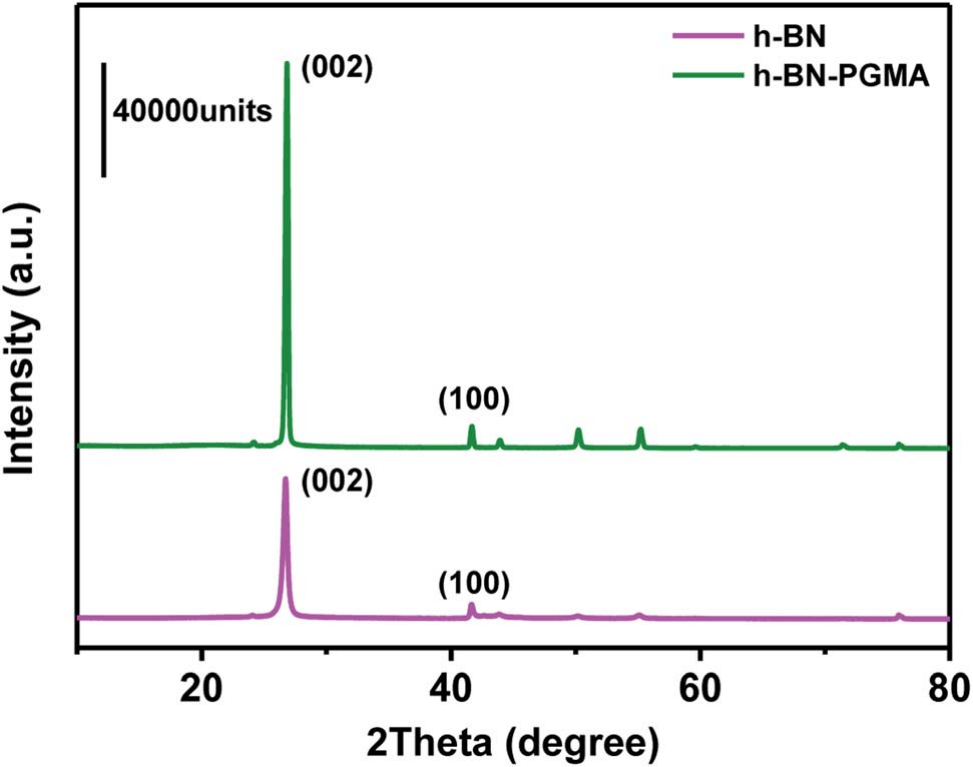
XRD patterns of h-BN and h-BN–PGMA composites.

To further demonstrate the orientation of h-BN–PGMA in the epoxy resin (EP) matrix, the h-BN orientation shown in Fig. 2 was investigated using XRD analysis. The h-BN–PGMA sheets in the horizontal and vertical directions were related to the (002) and (100) peaks, respectively.^21^ The difference in peak strength indicated that the orientation of the h-BN–PGMA sheet in the polymer matrix was different.^22^ The value of *α* was defined by the following formula, and the relative intensity of the (100) peak was compared with the sum of the relative intensities of (100) and (002) peaks.1
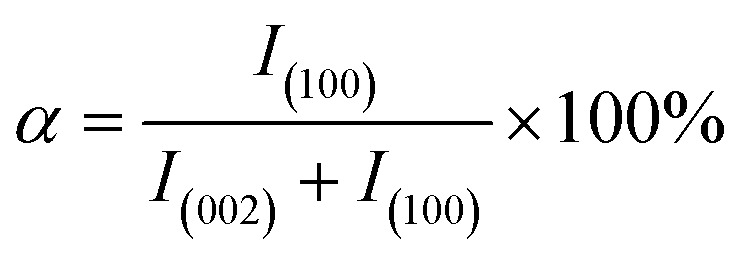


**Fig. 3:**
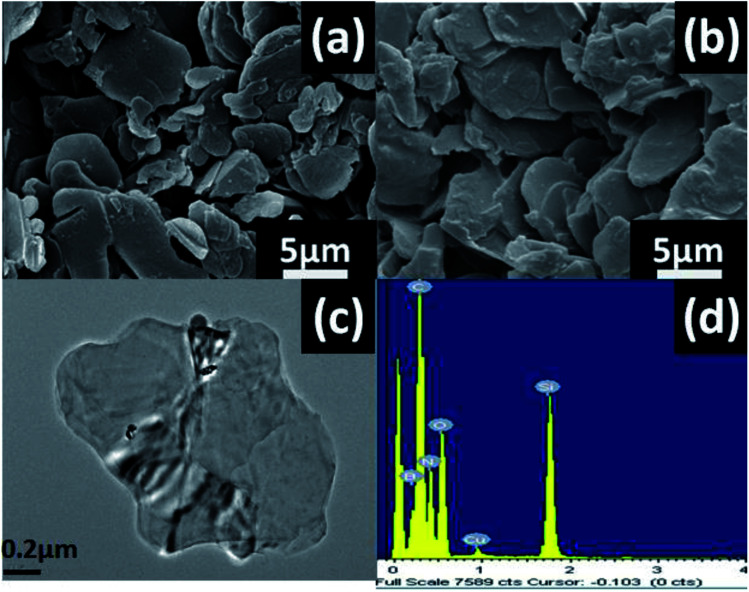
(a) SEM images of pure h-BN and (b) h-BN–PGMA, (c) TEM images of h-BN–PGMA and (d) EDS spectra of h-BN–PGMA.

According to Fig. 2 and formula (1), it is known that for the h-BN sheet, the value of *α* was 19.96%; for the h-BN–PGMA sheet, the value of *α* was 7.59%. As the filling amount of h-BN and h-BN–PGMA increased, the value of *α* increased. The lower value of *α* indicated that a large number of h-BN and h-BN–PGMA sheets were arranged in the in-plane direction. The results in Fig. 3(a) and (b) indicated that only a small amount of h-BN and h-BN–PGMA sheets accumulated in the direction of the through-plane direction.

Fig. 3 shows the morphology and elemental composition of h-BN and h-BN–PGMA, h-BN had a smooth surface with a size of 15.0 μm (Fig. 3(a)). The modified h-BN surface became rougher (Fig. 3(b)), which was due to the formation of a PGMA layer on the edge surface of h-BN sheets after the surface treatment (as shown in Fig. S1 and S2†). The EDS of h-BN–PGMA sheets showed higher contents of C and O elements (Fig. 3(d)). Which, indicated a PGMA layer was successfully grafted on the surface of the h-BN filler.^17^ TEM results showed a thin coating (a PGMA layer) on the h-BN filler surface (Fig. 3(c)). Such coating confirmed the successful grafting treatment of the surface of the h-BN sheet.

**Fig. 4:**
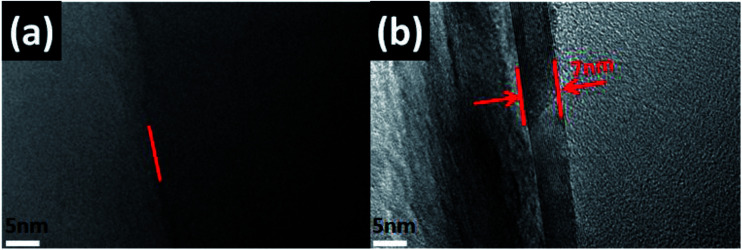
(a) and (b) TEM images of h-BN–PGMA microchip.

The TEM results shown in Fig. 4 indicated the contrast diagrams before and after the edge of boron nitride nano-sheets (BNNS) were grafted with polymer PGMA chains, Fig. 4(a) shows the morphology of BNNS before they were grafted with PGMA chains. The red arrow in Fig. 4(b) indicated that the thickness of the PGMA coating was about 7 nm, moreover, BNNS of 1 μm was attached to form a spherical BN structure by a PGMA chain (as shown in Fig. S1 and S2†), indicating that this coating was uniformly grafted to the edge of the h-BN sheets.

**Fig. 5:**
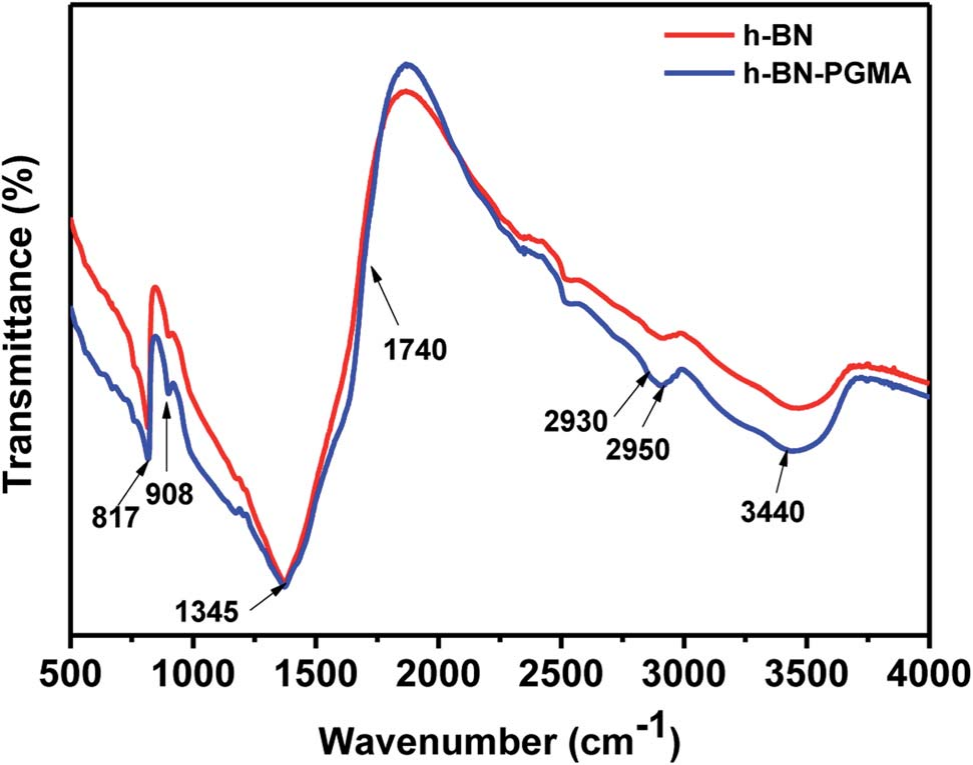
h-BN and h-BN–PGMA FTIR spectrum.

Fig. 5 shows FT-IR results on the surface modification. For h-BN sheets, peaks at 817 cm^−1^ and 1345 cm^−1^ were attributed to the B–N bond absorption peaks. Moreover, the peak at 3440 cm^−1^ might be caused by the dehydration of hydroxyl groups on BN fillers. While the ester peak of h-BN–PGMA sheets appeared at 1740 cm^−1^. Furthermore, the absorption peak of epoxides appeared at 908 cm^−1^. The peaks at 2930 cm^−1^ and 2950 cm^−1^ were attributed to the absorption vibration peaks of –CH_3_– and –CH_2_– bonds, respectively. FT-IR results showed that a PGMA layer had been successfully grafted onto the surface of h-BN sheet fillers.

**Fig. 6:**
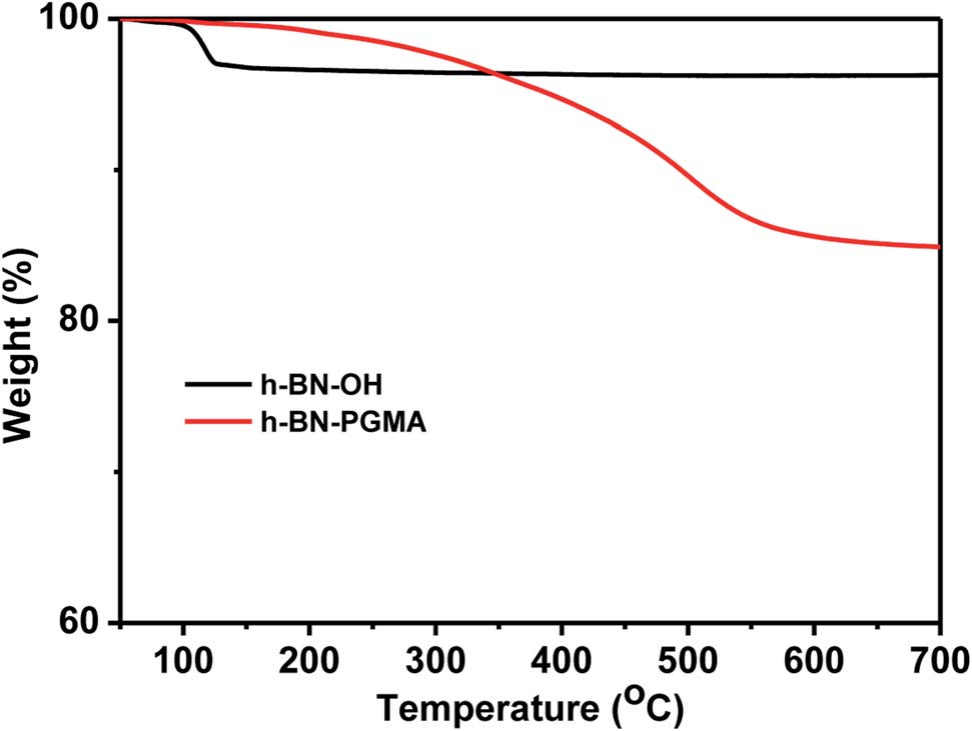
TGA curves of h-BN and h-BN–PGMA.

The TGA data in Fig. 6 show that the weight of the h-BN–PGMA sheets was obviously decreased. Nevertheless, because only a small amount of hydroxyl (–OH) was modified on the surface of h-BN sheets, the weight loss rate of the h-BN–PGMA was still very low. When the heating temperature was 120 °C, the weight loss rate of h-BN–OH was about 3%, which might be caused by the self-loss of some h-BN sheets during the heating process or the loss of water due to the hydroxyl reaction.^23^ The weight loss rate of h-BN–PGMA sheets was 17.7% at 700 °C, indicating that the PGMA chain was successfully attached to the surface of the h-BN sheets.

### 3.2 Dispersion and microstructure of h-BN–PGMA

**Fig. 7:**
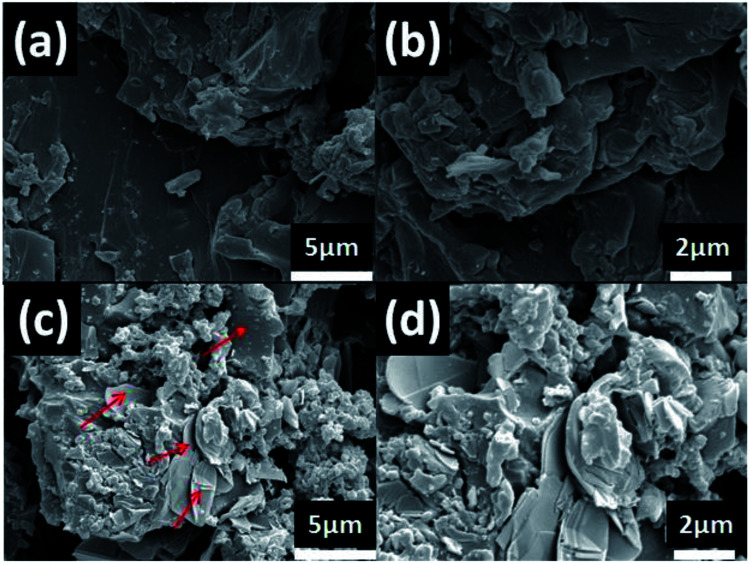
(a) The SEM image of the fracture surface of the h-BN composite with 16 vol%, (b) the magnified image of (a), (c) the SEM image of the fracture surface of the h-BN–PGMA composite with 16 vol%, (d) the magnified image of (c).

The dispersion state of the filler (h-BN–PGMA) was directly related to the thermal conductivity of the composite. By constructing the thermal conductivity network structure, strong interfacial adhesion between the filler and the matrix was obtained.^24^ To reveal the difference of any obvious interfacial interactions in these composites, the morphologies of different EP composites (Fig. 7) were observed by SEM. The h-BN sheets extended from the epoxy matrix, indicating that there were still some weak interfacial interactions in the epoxy composites (Fig. 7(b)). Nevertheless, h-BN–PGMA were implanting EP, and surrounded by absorbed EP (Fig. 7(c) and (d)), resulting in strong interfacial interactions in EP composites. There was no significant difference in the h-BN–PGMA dispersion state compared with h-BN. With 16 vol% of h-BN content (Fig. 7(a)) and h-BN–PGMA content (Fig. 7(c)), numerous h-BN or h-BN–PGMA microplatelets are seen in the field of vision connected to each other. Here, a distinct heat conduction network was constructed in the EP matrix.

### 3.3 Dynamic mechanical properties of composite materials

**Fig. 8:**
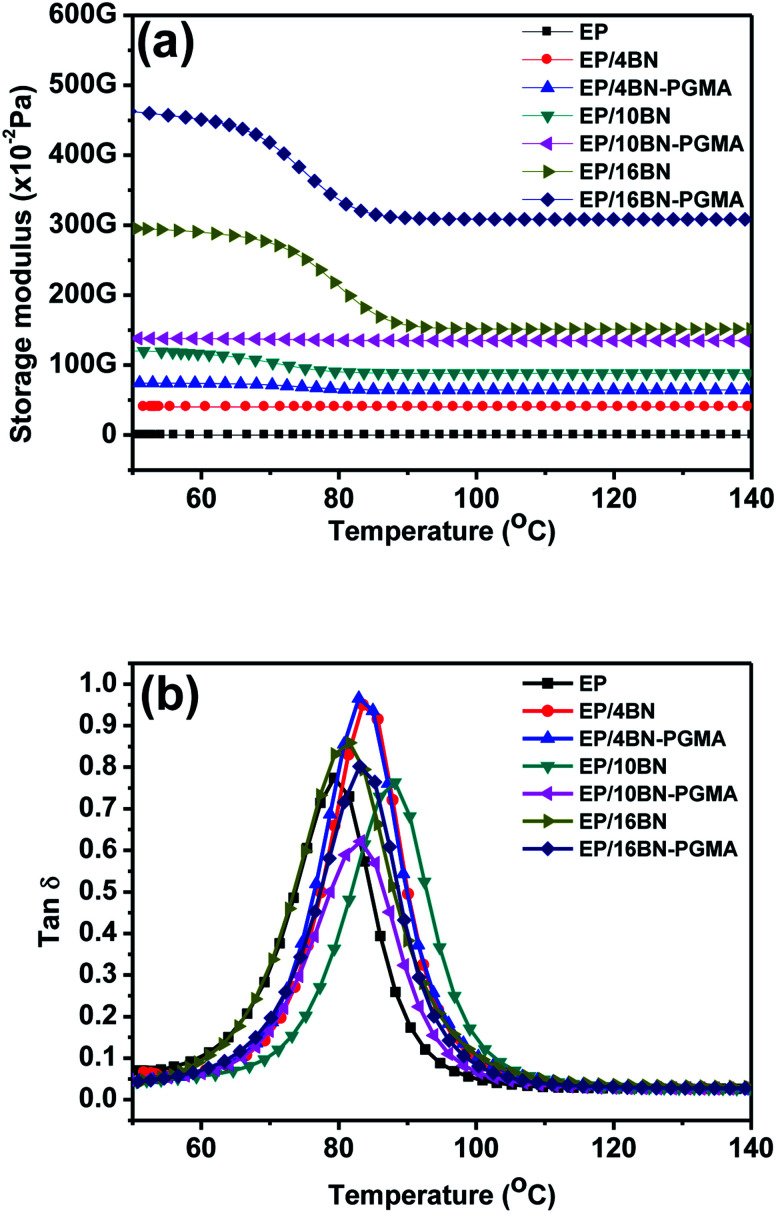
(a) Storage modulus and (b) loss coefficient (tan *δ*) of EP, EP/h-BN and EP/h-BN–PGMA composites.

Fig. 8(a) shows the storage modulus of the EP composite material. The stiffness of the filler allowed the stress with a greater degree to be transferred from the matrix to the filler. In addition, the graft-modified BN filler had a stronger interface-binding force and fewer defects than the BN filler without graft modification, so the EP/h-BN–PGMA composite had a high storage modulus. In Fig. 8(a), when the filling amount of h-BN–PGMA filler was 4 vol%, the storage modulus of EP/h-BN–PGMA composite material at 50 °C was 4.65 GPa, while the storage modulus of EP/h-BN composite materials (when h-BN was 4 vol%) at 50 °C was 2.95 GPa. This improvement was due to the interaction between the EP group on the h-BN edge and the EP matrix; therefore, enhanced interface adhesion could improve the transfer of external stress.^25^ The dielectric loss of the epoxy resin composite was obtained by DMA (Fig. 8(b)). The peak of the curve was the glass transition temperature (*T*_g_). When the filling amount was 16 vol%, the *T*_g_ of EP/h-BN–PGMA composite material increased from 79.2 °C of pure epoxy to 83.2 °C, and the *T*_g_ of EP/h-BN composite material increased from 79.2 °C of pure epoxy to 81.4 °C, indicating that the *T*_g_ had an equally upward trend. When the filling amount decreased to 4 vol%, the movement of the PGMA polymer segment was restricted by the interface between the matrix and the filler. Therefore, it relaxed its relaxation in the glass transition zone, resulting in an increase in *T*_g_.^26^ In general, the *T*_g_ value of composite materials with different amounts of h-BN–PGMA or h-BN was greater than the temperature value required by the electronic packaging primer.^27^

### 3.4 Thermal conductivity of composites

**Fig. 9:**
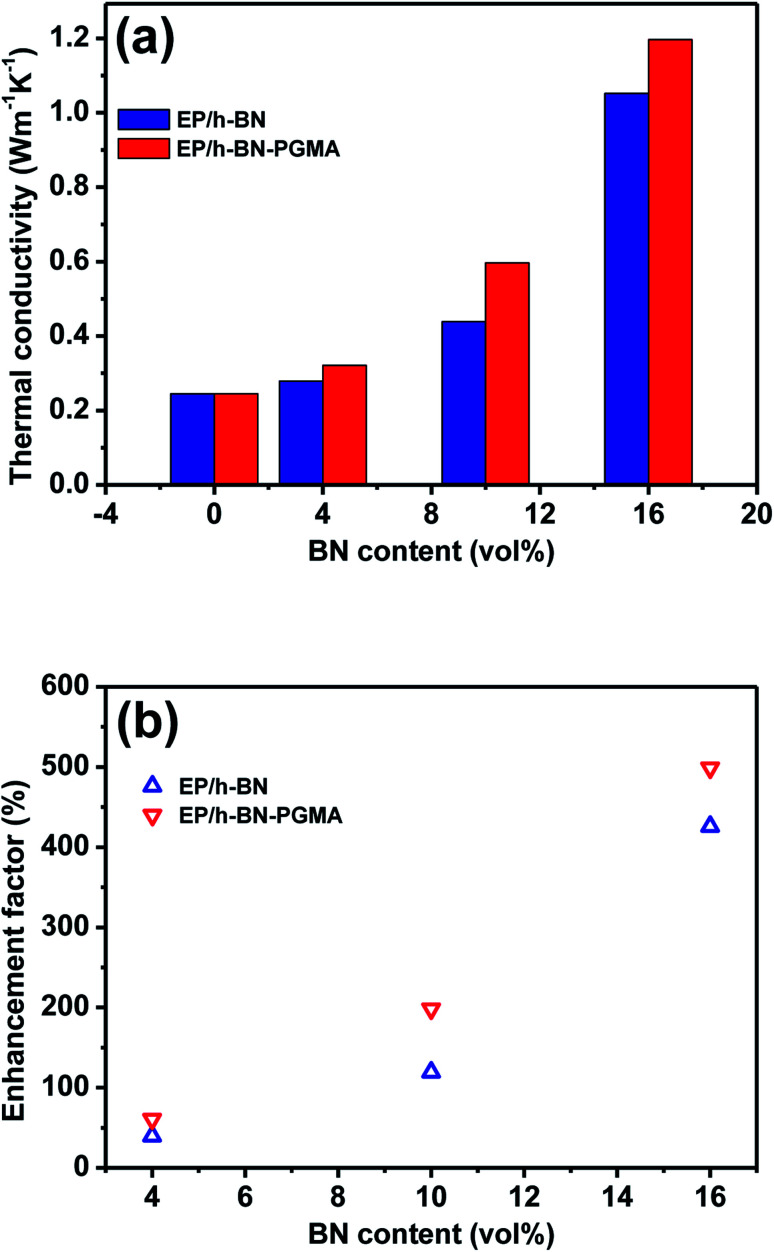
Thermal conductivity (a) thermal conductivity and (b) thermal conductivity enhancement ratio of pure EP and its composites.

Constructing thermal conduction channels and reducing interfacial thermal resistance are effective ways to improve the thermal conductivity of epoxy composites.^28^Fig. 9 shows the thermal conductivity of the composite materials. Fig. 9(a) shows that the thermal conductivity of the composite varied with the volume fraction of the filler. The thermal conductivity enhancement (*Φ*) of the composites and the data in Fig. 9(b) could be calculated and substituted using formula (2):2
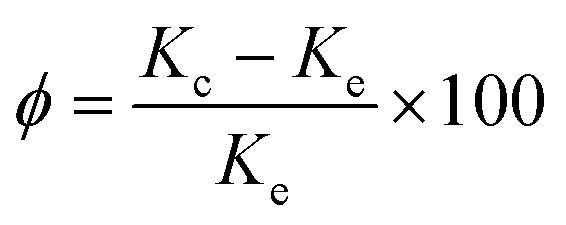
here, *K*_c_ and *K*_e_ represented the thermal conductivity of EP-based composite materials, and the thermal conductivity of pure EP resin materials, respectively. When the BN content increased from 4 vol% to 16 vol%, the thermal conductivity of EP/h-BN increased from 0.279 W m^−1^ K^−1^ to 1.052 W m^−1^ K^−1^, and the value of *Φ* increased from 40% to 426%. When the h-BN volume fraction-filling amount was increased from 10 vol% to 16 vol%, the *Φ* value was increased from 119% to 426%. This was because when the thermal conduction network was fully established,^29^ the thermal conduction increased rapidly. At low volume fractions, h-BN nanosheets were randomly embedded in EP matrix without contact. The increase of h-BN filling amount led to better connectivity of h-BN nanosheets, and the thermal conductivity of the composite material would also increase. From the experimental data, the graft modification on the filler surface could obviously improve the thermal conductivity of epoxy composites. When the volume fraction of h-BN–PGMA was 16 vol%, the thermal conductivity of the composite material was 1.197 W m^−1^ K^−1^, which was 4.99 times that of the pure EP resin. These results could be interpreted as PGMA had a longer molecular chain, leading to a stronger interface interaction.^30^ The PGMA macromolecular chain here acted as a bridge between h-BN nanosheets and epoxy resin (as shown in Fig. S4†). As confirmed from the transmission electron micrograph in Fig. 4, the stronger interface interaction could reduce the interface thermal resistance, resulting in higher thermal conductivity. There are two reasons for this: on the one hand, the PGMA macromolecular chain could enhance the compatibility and interface force between the filler and the matrix, but when the h-BN–PGMA filling amount exceeded 16 vol%, the thermal conductivity and dielectric properties of the composite material would decrease slightly (as shown in Table 1), making it difficult to meet the requirements of unfilled electronic packaging technology. For example, Zeng *et al.* developed an aerogel (3D-BNNS)/epoxy resin composite material, the results showed that the epoxy composite material with a low filler content (less than 10 vol%) had a high thermal conductivity (∼3.0 W m^−1^ K^−1^).^31^ The ordered arrangement of BN in the polymer matrix was the key to improving the thermal conductivity of the composite.^32^ Due to the high filler content and the single preparation method, it couldn't meet the requirements of low viscosity processes. Except for EP/BN/AgNPs composites,^33^ the thermal conductivity of epoxy composites made of epoxy resin and BN was generally lower than that of EP/h-BN–PGMA composites grafted by polymer links. However, there are few reports about EP/BN/AgNPs composite materials, and the filling of AgNPs would significantly increase the manufacturing cost of EP/BN composite materials, which has limited its application in the field of electronic packaging. In summary, the EP/h-BN–PGMA composite material with a filling amount of less than 18 vol% had a high thermal conductivity, which could meet the requirements of electronic packaging technology.

### 3.5 Dielectric properties of composite materials

**Fig. 10:**
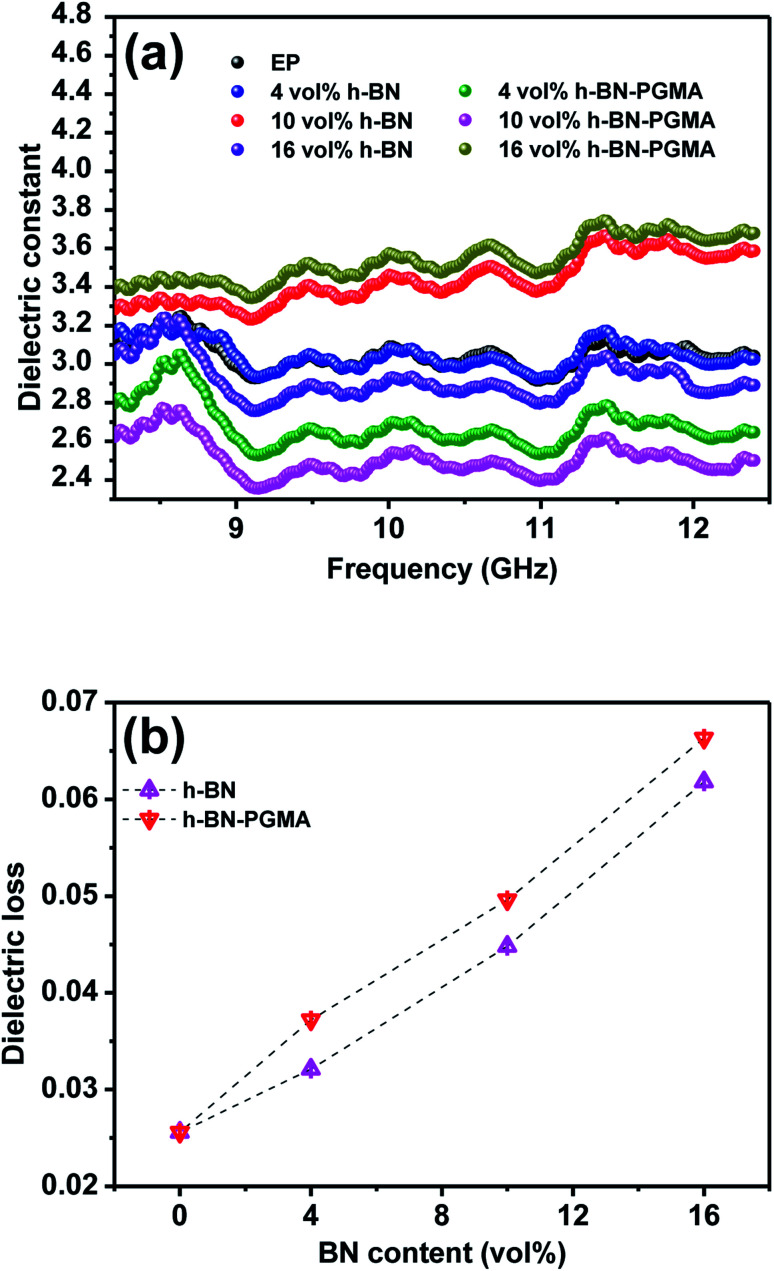
Dielectric properties of EP and composites: (a) frequency dependence of dielectric constant; (b) dielectric loss at 8.2 GHz.

The ideal dielectric properties are very important for unfilled electronic packaging processes. The importance of electronic packaging processes was to achieve an excellent operating speed of integrated devices.^34^Fig. 10 shows the dielectric properties of EP composites. It could be seen from Fig. 10(a) that the frequency was in the range of 8.2 GHz to 12.5 GHz. As the frequency increases, the dielectric constant of the composite material decreased slightly. For example, the dielectric constant of EP/h-BN–PGMA composite with 16 vol% filler at 8.2 GHz was 3.39, which was slightly higher than the dielectric constant of EP/h-BN composite with the same filling amount (*ε* = 3.05). This was because when PGMA was grafted on the surface of h-BN, the bond bridge formed between organic and inorganic materials increased the interfacial polarization and dielectric constant of the composite material.^35^Fig. 10(b) shows the tangent of the dielectric loss of the composite material at different filling levels at 8.2 GHz. As the filling amount increased, the dielectric loss of the composite material increased slightly. However, the EP/h-BN–PGMA composites had a lower dielectric constant and dielectric loss than the polymer composite reported in Table 1.

## 4. Conclusion

The grafted h-BN–PGMA/epoxy homogeneous dispersion composite material was prepared. This polymer link branch modified the thermal conductivity network not only enhanced the interface interaction between h-BN and EP but also improved the thermal conductivity of the composite material. The thermal conductivity of the EP/h-BN–PGMA composite material with a filling volume of 16 vol% was 1.197 W m^−1^ K^−1^, which was 4.99 times that of pure epoxy. At the same filling amount, the thermal conductivity of the EP/h-BN composite was lower than that of the EP/h-BN–PGMA composite. In summary, EP/h-BN–PGMA composite materials showed excellent insulation and thermal conductivity, ideal dielectric properties and low dielectric loss, and were used as candidates for the new generation of electronic packaging technology.

## Conflicts of interest

There are no conflicts to declare.

## Supplementary Material

RA-011-D1RA00976A-s001

## References

[cit1] Lu Y., Ballauff M. (2016). Spherical Polyelectrolyte Brushes as Nanoreactors for the Generation of Metallic and Oxidic Nanoparticles: Synthesis and Application in Catalysis. Prog. Polym. Sci..

[cit2] Cho H. B., Tokoi Y., Tanaka S., Suematsu H., Suzuki T., Jiang W., Niihara K., Nakayama T. (2011). Modification of BN Nanosheets and Their Thermal Conducting Properties in Nanocomposite Film with Polysiloxane According to the Orientation of BN. Compos. Sci. Technol..

[cit3] Zhang H. G. J. (2013). Ultra-flexible Polymethyl Methacrylate Composites Induced by Sliding of Micron-sized Hexagonal Boron Nitride Platelets. Ceram. Int..

[cit4] Liu Z., Li J., Liu X. (2020). Novel Functionalized BN Nanosheets/Epoxy Composites with Advanced Thermal Conductivity and Mechanical Properties. ACS Appl. Mater. Interfaces.

[cit5] Chen C., Xue Y., Li X., Wen Y., Liu J., Xue Z., Shi D., Zhou X., Xie X., Mai Y. W. (2019). High-performance Epoxy/binary Spherical Alumina Composite as Underfill Material for Electronic Packaging. Composites, Part A.

[cit6] Peng W., Huang X., Yu J., Jiang P., Liu W. (2010). Electrical and Thermophysical Properties of Epoxy/aluminum Nitride Nanocomposites: Effects of Nanoparticle Surface Modification. Composites, Part A.

[cit7] Narayan S. , Effect of Sonication Time, Concentration, Shape and Size of Nano Particle on Thermal Conductivity of Al_2_O_3_/water Nano Fluid, National Conference on Emerging Research Areas In Mechanical Engineering (ERAME – 2015), 2015

[cit8] Cao J.-P., Zhao X., Zhao J., Zha J.-W., Hu G.-H., Dang Z.-M. (2013). Improved Thermal Conductivity and Flame Retardancy in Polystyrene/poly(vinylidene fluoride) Blends by Controlling Selective Localization and Surface Modification of SiC Nanoparticles. ACS Appl. Mater. Interfaces.

[cit9] Baba S., Goto T., Cho S. H., Sekino T. (2018). Effect of Nitrogen Gas Pressure During Heat Treatment on the Morphology of Silicon Nitride Fibers Synthesized by Carbothermal Nitridation. Journal of Asian Ceramic Societies.

[cit10] Xiao G., Di J. T., Wang J. F. (2020). Highly Thermally Conductive, Ductile Biomimetic Boron Nitride/aramid Nanofiber Composite Film. Compos. Sci. Technol..

[cit11] Kwon O. H., Ha T., Kim D.-G., Kim B. G., Kim Y. S., Shin T. J., Koh W.-G., Lim H. S., Yoo Y. (2018). Anisotropy-driven High Thermal Conductivity in Stretchable Poly(vinyl alcohol)/Hexagonal Boron Nitride Nanohybrid Films. ACS Appl. Mater. Interfaces.

[cit12] Zhang H. (2015). Ultrathin Two-Dimensional Nanomaterials. ACS Nano.

[cit13] Yuan J., Qian X., Meng Z., Yang B., Liu Z.-Q. (2019). Highly Thermally Conducting Polymer-Based Films with Magnetic Field-Assisted Vertically Aligned Hexagonal Boron Nitride for Flexible Electronic Encapsulation. ACS Appl. Mater. Interfaces.

[cit14] Lin Y., Williams T. V., Connell J. W. (2010). Soluble, Exfoliated Hexagonal Boron Nitride Nanosheets. J. Phys. Chem. Lett..

[cit15] Sato K., Horibe H., Shirai T., Hotta Y., Nakano H., Nagai H., Mitsuishid K., Wataria K. (2010). Thermally Conductive Composite Films of Hexagonal Boron Nitride and Polyimide with Affinity-enhanced Interfaces. J. Mater. Chem..

[cit16] Chen J., Huang X., Zhu Y., Jiang P. (2017). Cellulose Nanofiber Supported 3D Interconnected BN Nanosheets for Epoxy Nanocomposites with Ultrahigh Thermal Management Capability. Adv. Funct. Mater..

[cit17] Zhao Y., Zhou M., Chen G., Zhou Z., Li Q. (2019). Hybridization of Polyhedral Oligomeric Silsesquioxane and Boron Nitride for Epoxy Composites with Improved Dielectric, Thermal and Tensile Properties. J. Mater. Sci..

[cit18] Tao H., Yao Y., Gang Z., Meng F. (2018). Silver Nanoparticle-Deposited Aluminum Oxide Nanoparticle as Fillers for Epoxy Composites with High Thermal Conductivity. Advanced Composites Letters.

[cit19] Pan C., Kou K. C., Jia Q., Zhang Y., Wu G. L., Ji T. Z. (2017). Improved Thermal Conductivity and Dielectric Properties of h-BN/PTFE Composites *via* Surface Treatment by Silane Coupling Agent. Composites, Part B.

[cit20] Gao B., Shi N., Shi X. (2013). Preparation of Grafted Particles PGMA/SiO_2_ with a New Surface-initiating System of Mercapto Group/BPO and Their Functionalization Transformation. J. Polym. Res..

[cit21] Lei Q. J., Gao B. J., Zhang Z. G. (2012). Preparation of Anion-type Bifunctional Carrier with Epoxy Group and Its Immobilization of Horseradish Peroxidase. Chin. J. Process Eng..

[cit22] Wang X., Wu P. (2019). 3D Vertically Aligned BNNS Network with Long-Range Continuous Channels for Achieving a Highly Thermally Conductive Composite. ACS Appl. Mater. Interfaces.

[cit23] Bhimanapati G. R., Kozuch D., Robinson J. A. (2014). Large-scale Synthesis and Functionalization of Hexagonal Boron Nitride Nanosheets. Nanoscale.

[cit24] Li X., Li Y., Alam M. M., Chen P., Xia R., Wu B., Qian J. (2020). Enhanced Thermal Conductivity of Nanocomposites with MOF-derived Encapsulated Magnetic Oriented Carbon Nanotube-grafted Graphene Polyhedra. RSC Adv..

[cit25] Liu Z., Li J., Liu X. (2020). Novel Functionalized BN Nanosheets/Epoxy Composites with Advanced Thermal Conductivity and Mechanical Properties. ACS Appl. Mater. Interfaces.

[cit26] Fang H., Bai S. L., Wong C. P. (2016). “White Graphene” – Hexagonal Boron Nitride Based Polymeric Composites and Their Application in Thermal Management. Compos. Commun..

[cit27] Xiao M., Du B. X. (2016). Review of High Thermal Conductivity Polymer Dielectrics for Electrical Insulation. High Voltage.

[cit28] Zhou W., Zhang Y., Wang J., Li H., Xu W., Li B., Chen L., Wang Q. (2020). Lightweight Porous Polystyrene with High Thermal Conductivity by Constructing 3D Interconnected Network of Boron Nitride Nanosheets. ACS Appl. Mater. Interfaces.

[cit29] Song N., Jiao D., Cui S., Hou X., Ding P., Shi L. (2017). Highly Anisotropic Thermal Conductivity of Layer-by-Layer Assembled Nanofibrillated Cellulose/Graphene Nanosheets Hybrid Films for Thermal Management. ACS Appl. Mater. Interfaces.

[cit30] Sun C., Zhao J., Zhang D., Guo H., Wang X., Hu H. (2020). Covalent Functionalization of Boron Nitride Nanosheets *via* Reductive Activation. Nanoscale.

[cit31] Zeng X., Yao Y., Gong Z., Wang F., Sun R., Xu J., Wong C. (2016). Ice-Templated Assembly Strategy to Construct 3D Boron Nitride Nanosheet Networks in Polymer Composites for Thermal Conductivity Improvement. Small.

[cit32] Geng Y., He H., Jia Y., Peng X., Li Y. (2019). Enhanced Through-Plane Thermal Conductivity of Polyamide 6 Composites with Vertical Alignment of Boron Nitride Achieved by Fused Deposition Modeling. Polym. Compos..

[cit33] Wang F., Zeng X., Yao Y., Sun R., Xu J., Wong C. P. (2016). Silver Nanoparticle-Deposited
Boron Nitride Nanosheets as Fillers for Polymeric Composites with High Thermal Conductivity. Sci. Rep..

[cit34] Chen J., Han J., Xu D. (2019). Thermal and Electrical Properties of the Epoxy Nanocomposites Reinforced with Purified Carbon Nanotubes. Mater. Lett..

[cit35] Tay R. Y., Li H., Tsang S. H., Jing L., Tan D., Wei M., Teo E. H. T. (2015). Facile Synthesis of Millimeter-Scale Vertically Aligned Boron Nitride Nanotube Forests by Template-Assisted Chemical Vapor Deposition. Chem. Mater..

