# Supplementary material for: Preparation of poly glycidyl methacrylate (PGMA) chain-grafted boron nitride/epoxy composites and their thermal conductivity properties
Source: RSC Adv. 2021 Jun 24;11(36):22343–51. doi: 10.1039/d1ra00976a (PMC9034223; doi:10.1039/d1ra00976a)
Supplement: RA-011-D1RA00976A-s001 [file RA-011-D1RA00976A-s001.pdf]

Electronic Supplementary Material (ESI) for RSC Advances.
This journal is © The Royal Society of Chemistry 2021

Supporting Information

Preparation of PGMA Chain-grafted Boron Nitride/Epoxy Composites and Their Thermal Conductivity Properties

Haibao Zhang,[a,b,*] Xian Zhang,[a,b] Kang Zheng,[a,b] Xingyou Tian[a,b,*]

[a]Institute of Solid Physics, Hefei Institutes of Physical Science, Chinese Academy of Sciences, Hefei, People's Republic of China

[b]Key Laboratory of Photovolatic and Energy Conservation Materials, Chinese Academy of Sciences, Hefei, People's Republic of China

.[*] To whom all correspondence should be addressed (E-mail: hbzhang@rntek.cas.cn, xytian@issp.ac.cn)

Figure S1. Haibao Zhang et al

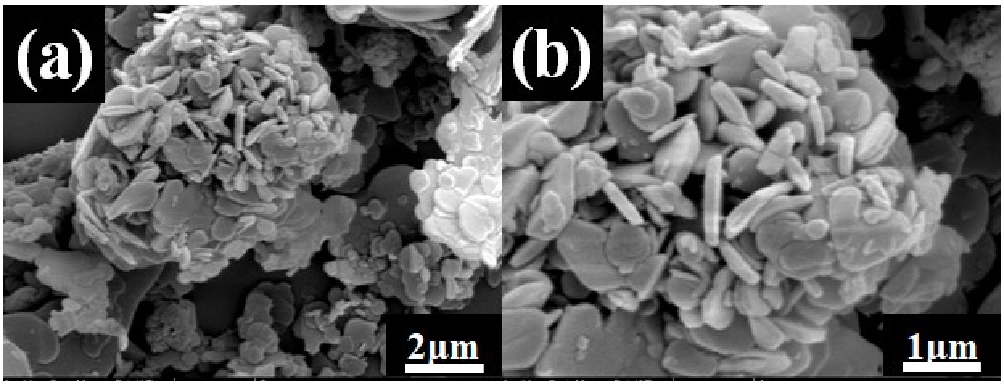

Figure S1. SEM images of the BNNS grafted by PGMA chain

Figure S2. Haibao Zhang et al

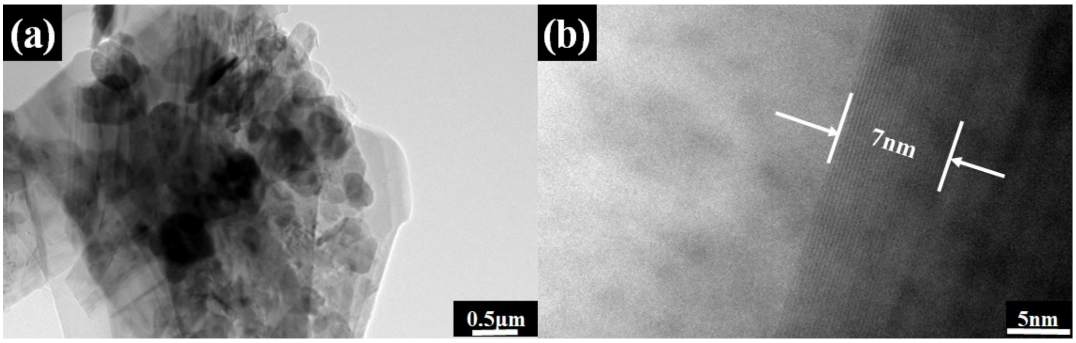

Figure S2. TEM image (a) and HRTEM image (b) of the BNNS grafted by PGMA chain, white mark is the thickness of the grafted PGMA chain

Figure S3. Haibao Zhang et al

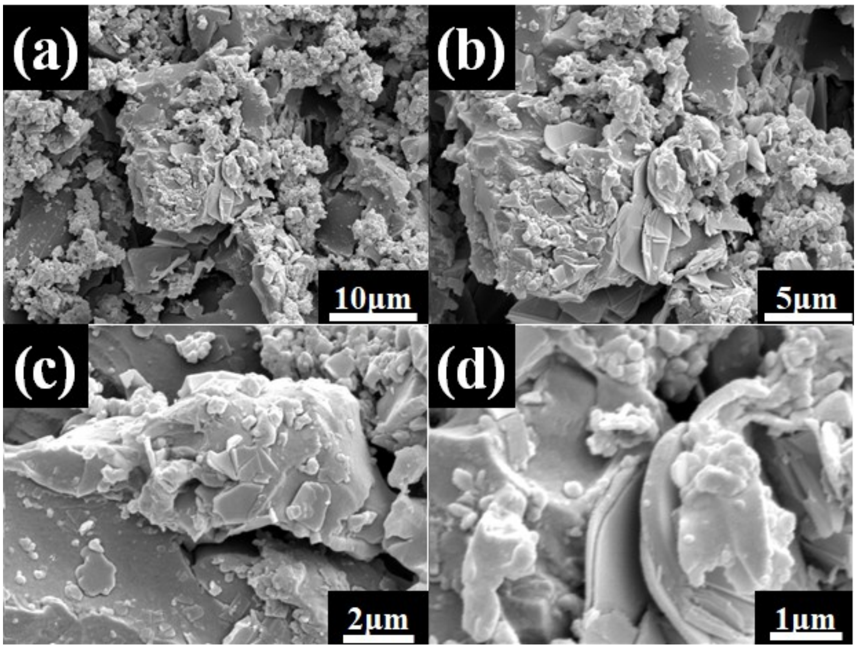

Figure S3. The SEM images of the 3 um-15um compound BNNS grafted by PGMA chain

Figure S4. Haibao Zhang et al

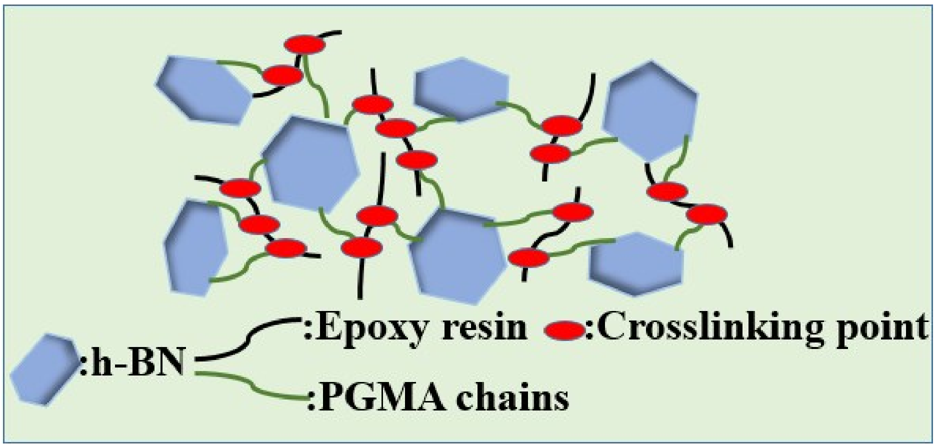

Figure S4. Bridging action of PGMA chains between h-BN and epoxy resin
